# Sofosbuvir-based direct-acting antivirals and changes in cholesterol and low density lipoprotein-cholesterol

**DOI:** 10.1038/s41598-022-13657-5

**Published:** 2022-06-15

**Authors:** Yi-Kai Wang, Ying-Wen Wang, Chia-Ling Lu, Yi-Hsiang Huang, Ming-Chih Hou, Yuh-Lih Chang, Wei-Ping Lee, Keng-Hsin Lan

**Affiliations:** 1 Division of Gastroenterology and Hepatology, Department of Medicine, Taipei Veterans General Hospital, Taipei, Taiwan; 2 Department of Medical Research, Taipei Veterans General Hospital, Taipei, Taiwan; 3 Healthcare Center, Taipei Veterans General Hospital, Taipei, Taiwan; 4 School of Medicine, National Yang Ming Chiao Tung University, Taipei, Taiwan; 5 Institute of Pharmacology, College of Medicine, National Yang Ming Chiao Tung University, Taipei, Taiwan; 6 Institute of Biochemistry and Molecular Biology, College of Life Sciences, National Yang Ming Chiao Tung University, Taipei, Taiwan; 7 Institute of Clinical Medicine, College of Medicine, National Yang Ming Chiao Tung University, Taipei, Taiwan; 8 Department of Pharmacy, Taipei Veterans General Hospital, Taipei, Taiwan; 9 Department of Pharmacy, College of Pharmaceutical Sciences, National Yang Ming Chiao Tung University, Taipei, Taiwan; 10 Department of Pharmacy, National Yang Ming Chiao Tung University Hospital, Yilan, Taiwan

**Keywords:** Diseases, Gastroenterology, Medical research

## Abstract

Worsened lipid profiles were observed in chronic hepatitis C (CHC) patients during direct-acting antivirals (DAAs) treatment, among which combination drugs confounded the effect of individual ingredient on lipid. Tenofovir alafenamide (TAF) also worsened lipid profiles in HIV patients. Structural similarity between sofosbuvir (SOF) and TAF prompted us to investigate rapid increase in total cholesterol (TC) and low-density lipoprotein cholesterol (LDL-C) in CHC patients treated with SOF-based DAAs. A retrospective study was performed to analyze 487 CHC patients receiving DAAs with SVR12. Relative risks on elevating TC and LDL-C were analyzed by logistic regression to determine SOF-based over non-SOF-based regimens. TC or LDL-C levels at baseline, week-4 and SVR12 were compared by Wilcoxon matched-pairs signed rank test. Week 4 or SVR12 to baseline ratios of serum TC or LDL-C between regimens were compared by Mann–Whitney's test. 487 patients were treated with Harvoni (SOF-based, 206 patients), Epclusa (SOF-based, 124 patients), Maviret (non-SOF-based, 122 patients), or Zepatier (non-SOF-based, 35 patients). At week 4 during drug treatment, Harvoni, Epclusa, and Maviret induced statistically significant elevation of TC and LDL-C, but Zepatier did not. SOF-based regimens had 2.72-fold higher relative risk (RR) causing 10% elevation of TC (95% CI 1.84–4.02, *p* < 0.001) and 2.04-fold higher RR causing 10% elevation of LDL-C (95% CI 1.39–3.01, *p* < 0.001) than non-SOF-based DAAs. SOF-based DAAs were associated with significantly larger amplitude of increases in TC and LDL-C than non-SOF-based DAAs during the initial 4 weeks of treatment, but the increases were not sustained to SVR12.

## Introduction

Hepatitis C virus (HCV) is one of the major causes of liver-related morbidity and mortality^1^, estimating infection of approximately 180 million people worldwide^2^. The HCV life cycle is initiated by binding of virus particles to hepatocellular receptors, endocytosis, fusion of HCV glycoproteins with endosomal membranes, acidification of endosome, and release of the viral genome into cytosol for replication^3^. Internal ribosome entry site (IRES)-mediated translation of incoming viral RNA enables viral gene expression and processing, and replication occurs in the “membranous webs”^4^. Following replication, genomic RNAs in complex with NS5A protein (Nonstructural protein 5A) transit to lipid droplets, where core protein localizes and virion assembly occurs^5^. After acquiring apolipoproteins B and E (apoB and apoE), components of VLDL (very low-density lipoproteins) and LDL (low-density lipoprotein), HCV infectious particles egress in a manner that parallels the VLDL secretory pathway^6,7^. HCV-infected patients have a prevalence of hepatic steatosis twofold higher than in HBV (Hepatitis B virus)-infected patients^8,9^, demonstrating a clear correlation between HCV infection and non-alcoholic fatty liver disease. These patients are also more likely to present decreased serum levels of apoB-bearing lipoproteins because HCV seizes these lipoproteins^10,11^.

HCV nonstructural proteins 4B, 5A and 5B constitute a complex for RNA replication. The RNA translates into a polyprotein processed by viral NS3/4A and host proteases to generate structural proteins for viral assembly and nonstructural proteins involved in RNA replication^12^. Currently, direct-acting antivirals (DAAs), which have replaced interferon, are the standard treatment for HCV infection^13,14^. According to mechanisms of action and therapeutic targets, DAAs are classified into four categories: NS3/4A protease inhibitor, NS5A replication complex inhibitor, and NS5B nucleoside and non-nucleoside polymerase inhibitor^15^. In the class of NS5B nucleoside polymerase inhibitor, sofosbuvir (SOF) is the only drug and plays an important role in the combination of other DAAs for HCV treatment^13,14^.

Although DAAs provide well-tolerated, safe, and highly efficacious outcomes^16^, several studies reported worsened lipid profiles in chronic hepatitis C (CHC) patients during DAAs treatment^17,18^. SOF-based regimens appeared to have greater effect on low density lipoprotein-cholesterol (LDL-C) elevation. In the study by Meissner et al., the patients treated with SOF/ribavirin had significantly increased levels (LDL-C) from baseline to the end of treatment and to post-treatment week 48^17^. Younossi et al. also found significantly increased LDL-C from baseline to the end of treatment and to post-treatment week 4 in CHC patients treated with SOF/ledipasvir^18^.

**Figure 1:**
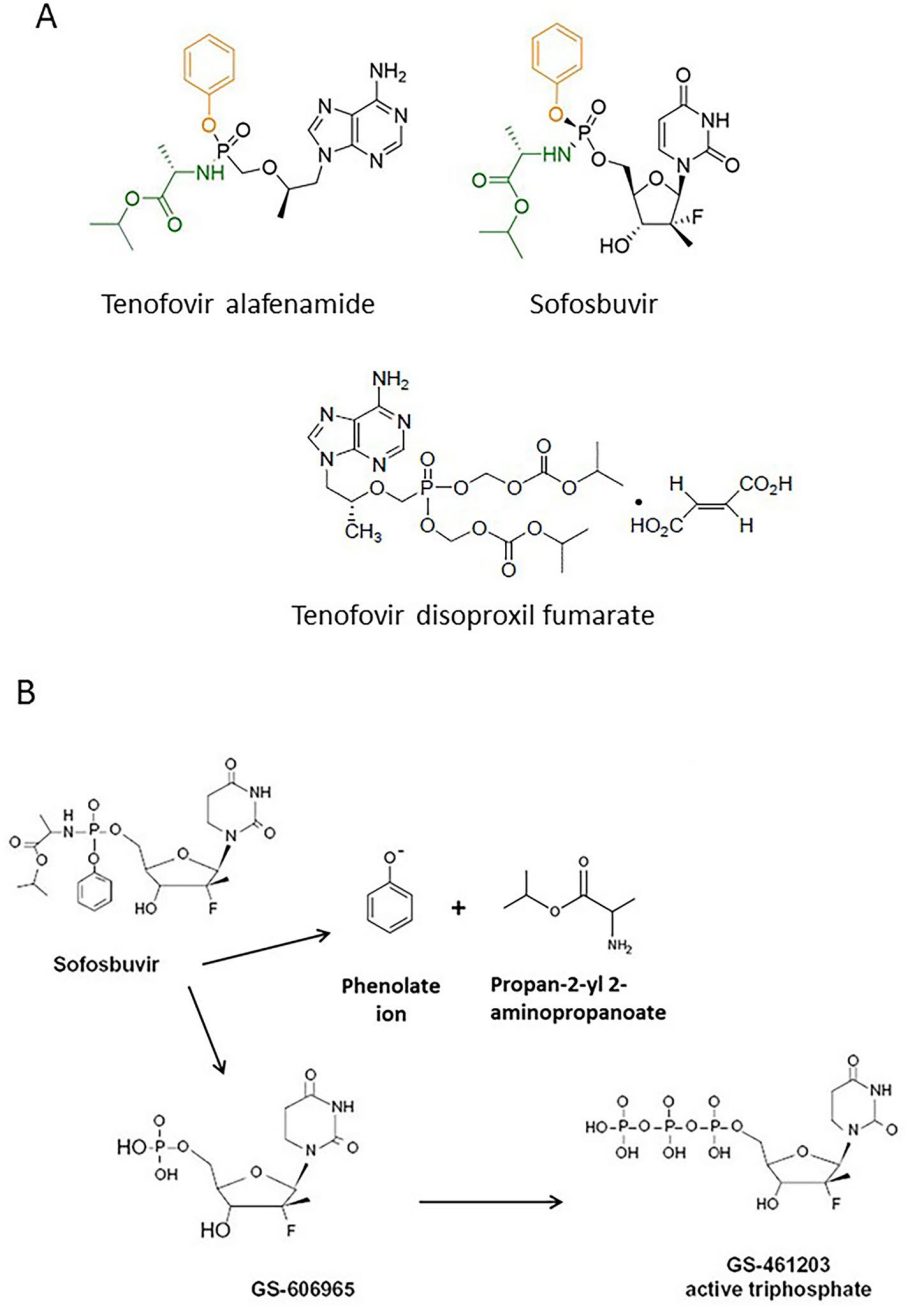
Structures of tenofovir alafenamide (TAF), sofosbuvir (SOF), and tenofovir disoproxil fumarate (**A**). TAF and SOF have similar phosphoramidate side chain (green and orange in the figure) that is cleaved from sofosbuvir to produce an active nucleotide analogue, GS-461203 (2′-deoxy-2′-α-fluoro-β-C-methyluridine-5′-triphosphate) (**B**).

HCV virion is tightly associated with hepatocyte-derived lipoproteins to form a lipid-laden particle, called lipo-viro-particle. It was thought that HCV hijacks lipoproteins that are released to the blood after clearance of the virus by DAAs. Given that the concept is true, all DAA regimens would have equivalent effects on lipid profiles of the blood. In this report, we performed retrospective analysis from the laboratory data of DAA-treated CHC patients based on structural similarity between TAF (tenofovir alafenamide) and SOF (Fig. 1A) and the observation that patients given TAF for HIV (human immunodeficiency virus) infection had elevated cholesterol level^19–21^. Both TAF and SOF are prodrugs with phosphoramidate and phosphoxybenzene side chains that were enzymatically cleaved in the cell to produce bioactive nucleotide analogues by releasing phenolate ion and propan-2-yl 2-aminopropanoate^22,23^ Therefore, we hypothesized that SOF might be the major contributor to total cholesterol (TC) and LDL-C elevations during DAAs treatment for patients infected by HCV. The mechanism may be related to the phosphoramidate side chain that is cleaved from sofosbuvir to produce an active nucleotide analogue, GS-461203 (2′-deoxy-2′-α-fluoro-β-C-methyluridine-5′-triphosphate)^24^ (Fig. 1B). To prove our hypothesis, we included HCV patients treated with SOF- or non-SOF-based DAAs and compared lipid profiles between the two groups during and after treatment. We found that SOF-based regimens contributed more to elevated TC and LDL-C during treatment, however, the elevations returned to baseline at SVR12 (sustained virologic response 12 weeks post-treatment).

## Methods

### Study population

The study retrospectively enrolled HCV-infected patients who received DAAs treatment for 8 or 12 weeks at Taipei Veterans General Hospital from September 2018 to August 2020. All patients were aged above 18, male or female and had chronic HCV infection, defined as detectable anti-HCV antibody and HCV RNA level in the serum for more than 6 months. Patients were excluded from the study if they had decompensated cirrhosis. The study was approved by the Institutional Review Board of Taipei Veterans General Hospital and was conducted in accordance with the principles of Declaration of Helsinki and the International Conference on Harmonization for Good Clinical Practice. All patients read and signed informed consent before drug prescription and study-related procedure.

### Study design

All patients tested positive for anti-HCV antibody in the blood underwent a series of blood tests as shown in Table 1 including HCV genotype and HCV viral loads. Those with positive HCV RNA in the blood were treated with DAAs. All patients’ daily medications were subjected to drug-drug interaction screening for DAAs. Lipid-lowering medications were discontinued during DAAs treatment and 12 weeks post-treatment. Baseline demographic data were collected before treatment. Hemogram, serum biochemical profiles (albumin, total bilirubin, direct bilirubin, aspartate aminotransferase [AST], alanine aminotransferase [ALT], creatinine, international normalized ratio [INR], estimated glomerular filtration rate [eGFR]), anti-HCV, hepatitis B virus surface antigen (Abbott Architect HBsAg qualitative assay, Abbott Laboratories), HCV RNA and HCV genotype (Abbott RealTime HCV Genotype II, Abbott Laboratories, Abbott) were obtained from all patients. Hemogram and serum biochemistry were collected at baseline, week 4 and SVR12. Non-cirrhotic patients were treated with DAAs as suggested in package insert, with or without with weight-based ribavirin (RBV, Robatrol, 200 mg capsule, Genovate Biotechnology Co., Ltd. Taiwan; 1200 mg daily if the body weight ≥ 75 kg; 1000 mg daily if the body weight < 75 kg) for 8 or 12 weeks. The DAA regimens included SOF/LED (sofosbuvir 400 mg/ledipasvir 90 mg, Harvoni, 206 patients), SOF/VEL (sofosbuvir 400 mg/velpatasvir 100 mg, Epclusa, 124 patients), GLE/PIB (glecaprevir 100 mg/pibrentasvir 40 mg**,** Maviret, 122 patients), and ELB/GRA (elbasvir 50 mg/grazoprevir 100 mg, Zepatier, 35 patients). Eight patients took RBV for 12 weeks with SOF/VEL, and 7 patients took RBV for 12 weeks with SOF/LED (Table 1).

**Table 1:** Characteristics of patients.

	All patients (n = 487)	Patients with SOF-based regimen (n = 330)	Patients with non-SOF-based regimen (n = 157)	*p*^a^
Male, (%)	247 (50.7)	160 (48.5)	87 (55.4)	0.153
Age, median (range)	63 (18–96)	62 (18–96)	64 (23–96)	0.913
Age < 55 years, (%)	138 (28.3)	92(27.9)	46 (29.3)	0.745
**HCV genotype, (%)**				
1a	35 (7.2)	26 (7.9)	9 (5.7)	0.456
1b	255 (52.4)	177 (53.6)	78 (49.7)	0.414
2	160 (32.9)	104 (31.5)	56 (35.7)	0.362
3	5 (1)	1 (0.3)	4 (2.6)	0.039
6	20 (4.1)	15 (4.6)	5 (3.2)	0.627
Unknown	12 (2.5)	7 (2.1)	5 (3.2)	0.536
Treatment-naïve, (%)	451 (92.6)	305 (92.4)	146 (93)	1.000
**DAA regimen**				
SOF-based regimen (n = 330)	330 (67.8)	330 (100)		
SOF/VEL (n = 124)	124 (25.5)	124 (37.6)		
For 12 weeks, (%)	116 (23.8)	116 (35.2)		
For 12 weeks with RBV, (%)	8 (1.6)	8 (2.4)		
SOF/LED (n = 206)	206 (42.3)	206 (62.4)		
For 8 weeks, (%)	3 (0.6)	3 (0.9)		
For 12 weeks, (%)	196 (40.2)	196 (59.4)		
For 12 weeks with RBV, (%)	7 (1.4)	7 (2.1)		
Non-SOF-based regimen (n = 157)	157 (32.2)		157(100)	
GLE/PIB (n = 122)	122 (25.1)		122 (77.7)	
For 8 weeks, (%)	110 (22.6)		110 (70.1)	
For 12 weeks, (%)	12 (2.5)		12 (7.6)	
ELB/GRA (n = 35)	35 (7.2)		35 (22.3)	
For 12 weeks, (%)	35 (7.2)		35 (22.3)	
HBsAg positivity, (%)	35 (7.2)	26 (7.9)	9 (5.7)	0.456
Anti-HIV positivity, (%) (n = 477)	41/477 (8.6)	22/327 (6.7)	19/150 (12.7)	0.036
Hepatocellular carcinoma, (%)	40 (8.2)	23 (7)	17 (10.8)	0.160
Other malignancy, (%)	43 (8.8)	25 (7.6)	18 (11.5)	0.173
BMI, kg/m^2^, median (range) (n = 420)	23.8 (15.8–36.7)	23.9 (15.8–36.7)	23.8 (17.1–33.8)	0.638
BMI < 30 kg/m^2^, (%)	383/420 (91.2)	249/277 (89.9)	134/143 (93.7)	0.209
White blood cell count, 10^9^ cells/L, median (range) (n = 486)	5.4 (1.65–13)	5.3 (2.1–11.9)	5.65 (1.65–13)	0.028
Hemoglobin level, g/dL, median (range) (n = 486)	13.6 (5.3–18.4)	13.5 (5.3–18.4)	13.8 (7.6–17.6)	0.734
Platelet count, 10^9^ cells/L, median (range)	176 (24–598)	173 (24–516)	185 (33–598)	0.006
INR, median (range) (n = 486)	1.04 (0.85–2.51)	1.04 (0.85–2.39)	1.05 (0.89–2.51)	0.539
Total cholesterol, mg/dL, median (range)	162 (32–319)	162 (32–295)	162 (54–319)	0.950
LDL-C, mg/dL, median (range)	95 (29–222)	95 (29–185)	95 (37–222)	0.649
Albumin, g/dL, median (range)	4.2 (1.9–6.8)	4.2 (1.9–6.8)	4.3 (2.7–5)	0.306
Total bilirubin, mg/dL, median (range)	0.68 (0.15–6.3)	0.7 (0.15–5.2)	0.68 (0.16–6.3)	0.084
Direct bilirubin, mg/dL, median (range) (n = 481)	0.29 (0.07–4.8)	0.3 (0.07–4.8)	0.27 (0.09–0.99)	0.002
AST, ULN, median (range) (n = 485)	43 (11–711)	46 (11–711)	38 (12–298)	0.003
ALT, ULN, median (range) (n = 486)	51 (2–1125)	52 (2–1125)	48 (8–737)	0.088
Creatinine, mg/dL, median (range) (n = 486)	0.83 (0.3–14.7)	0.8 (0.3–12.3)	0.9 (0.43–14.7)	< 0.001
eGFR, mL/min/1.73 m^2^, median (range)	82 (3–186)	83 (4–186)	80 (3–157)	0.017
eGFR ≥ 60 mL/min/1.73 m^2^, (%)	403 (82.8)	287 (87)	116 (73.9)	< 0.001
Hemodialysis, (%)	24 (4.9)	9 (2.7)	15 (9.6)	0.003
HCV RNA level, log_10_ IU/mL, median (range)	6.18 (1.76–8.2)	6.16 (1.76–7.67)	6.25 (2.71–8.2)	0.319
HCV RNA level < 800,000 IU/mL, (%)	196 (40.3)	135 (40.9)	61 (38.9)	0.665
HCV RNA level < 6,000,000 IU/mL, (%)	358 (73.5)	250 (75.8)	108 (68.8)	0.103
**Stage of hepatic fibrosis by FIB-4 (n = 485)**				
F0, (%)	115/485 (23.6)	69/328 (20.9)	46/157 (29.3)	0.052
F1, (%)	115/485 (23.6)	75/328 (22.7)	40/157 (25.5)	0.496
F2, (%)	100/485 (20.5)	70/328 (21.2)	30/157 (19.1)	0.633
F3, (%)	101/485 (20.7)	70/328 (21.2)	31/157 (19.8)	0.811
F4, (%)	54/485 (11.1)	44/328 (13.3)	10/157 (6.4)	0.021

Continuous variables are shown as median (with range) analyzed by Mann–Whitney's test. Categorical variables are expressed as number of patients (n) with frequencies (%) analyzed by Chi-squared test and Fisher’s exact test.

ALT: alanine aminotransferase, AST: aspartate aminotransferase, BMI: body mass index, DAA: direct-acting antiviral, eGFR: estimated glomerular filtration rate, ELB/GRA: elbasvir/grazoprevir, GLE/PIB: glecaprevir/pibrentasvir, HBsAg: hepatitis B virus surface antigen, HCV: hepatitis C virus, HIV: human immunodeficiency virus, INR: international normalized ratio, LDL-C: low-density lipoprotein-cholesterol, SOF/LED: sofosobuvir/ledipasvir, RNA: ribonucleic acid, ULN: upper limit of normal, SOF/VEL: sofosbuvir/velpatasvir.

^a^SOF-based regimen v.s non-SOF-based regimen.

### Virologic assessment

On-treatment effectiveness was assessed by detecting serum HCV RNA levels at weeks 4 and 12 (there was no week 8 data). The effectiveness at the end of treatment was SVR12, defined as serum HCV RNA level < LLOQ (lower limit of quantification) 12 weeks after completed treatment.

### Statistical analyses.

All analyses were performed using STATA (12th ed., developed by StataCorp LLC, College Station, TX, USA). The pre-treatment patient characteristics were shown in median (range) and percentages as appropriate and compared by Mann–Whitney's test and χ^2^ with Fisher’s exact test. The effectiveness of treatment during and after drug administration was shown in number and percentages. Relative risks on elevating TC and LDL-C were analyzed by logistic regression to determine SOF-based regimens over non-SOF-based regimens. TC or LDL-C levels at baseline, week 4 and SVR12 were compared by Wilcoxon matched-pairs signed rank test. Week 4 or SVR12 to baseline ratios of serum TC or LDL-C between regimens were compared by Mann–Whitney's test. All statistics were two-tailed, and the results were considered statistically significant when a *p* value was < 0.05.

### Consent to participate

The study was approved by the Institutional Review Board of Taipei Veterans General Hospital and was conducted in accordance with the principles of Declaration of Helsinki and the International Conference on Harmonization for Good Clinical Practice. All patients read and signed informed consent before drug prescription and study-related procedure.

### Consent to publish

All authors agree that the copyright is transferred to the journal in case of acceptance of the manuscript.

## Results

### Patient characteristics

Four hundred and eighty-seven CHC patients achieving SVR12 were included in the study (Table 1). The median age was 63 years, and 247 patients were male (50.7%). The two major genotypes (GT) are GT 1b (255 patients, 52.4%) and GT 2 (160 patients, 32.9%). Four hundred and fifty-one patients were treatment-naïve (92.6%), and 35 had HBV co-infection (7.2%). No patient received antiviral therapy for HBV, and neither developed HBV reactivation nor hepatitis flares during DAA therapy. Forty-one patients had HIV co-infection, who took anti-HIV drugs. The median log_10_ HCV RNA level was 6.18. One hundred and ninety-six patients had a baseline viral load < 800,000 IU/mL (40.3%). One hundred and one patients had a Fib-4 fibrosis stage of F3 (20.7%), and 54 patients, F4 (11.1%) (Table 1). The patient percentages of F0, F1, F2, and F3 were similar between SOF and non-SOF groups. F4 fibrosis is 13.3% in SOF group and 6.4% in non-SOF group.

### Effectiveness

Four hundred and ninety-five patients were treated with different DAA regimens. Four hundred and eighty-seven patients achieved SVR12. The SVR12 rates for SOF/VEL, SOF/LED, GLE/PIB, and ELB/GRA were 100% (124/124), 99.5% (206/207), 94.6% (122/129), and 100% (35/35), respectively (Table 2).

**Table 2:** Viral responses to different DAA regimens (n = 495).

DAA regimens	Serum HCV RNA < LLOQ, SVR12, no	Failed SVR12, no	Missing data to SVR12, no
**Sofosbuvir-based regimen (n = 331)**	330 (99.7%)	1 (0.3%)	
SOF/VEL (n = 124)	124 (100%)		
SOF/LED (n = 207)	206 (99.5%)	1 (0.5%)	
**Non-sofosbuvir-based regimen (n = 164)**	157 (95.7%)	5 (3%)	2 (1.2%)
GLE/PIB (n = 129)	122 (94.6%)	5 (3.9%)	2 (1.6%)
ELB/GRA (n = 35)	35 (100%)		

DAA: direct-acting antiviral, ELB/GRA: elbasvir 50 mg and grazoprevir 100 mg, GLE/PIB: glecaprevir 100 mg pibrentasvir 40 mg, SOF/LED: sofosobuvir 400 mg and ledipasvir 90 mg, SOF/VEL: sofosbuvir 400 mg and velpatasvir 100 mg.

### Changes in TC and LDL-C at week 4

**Figure 2:**
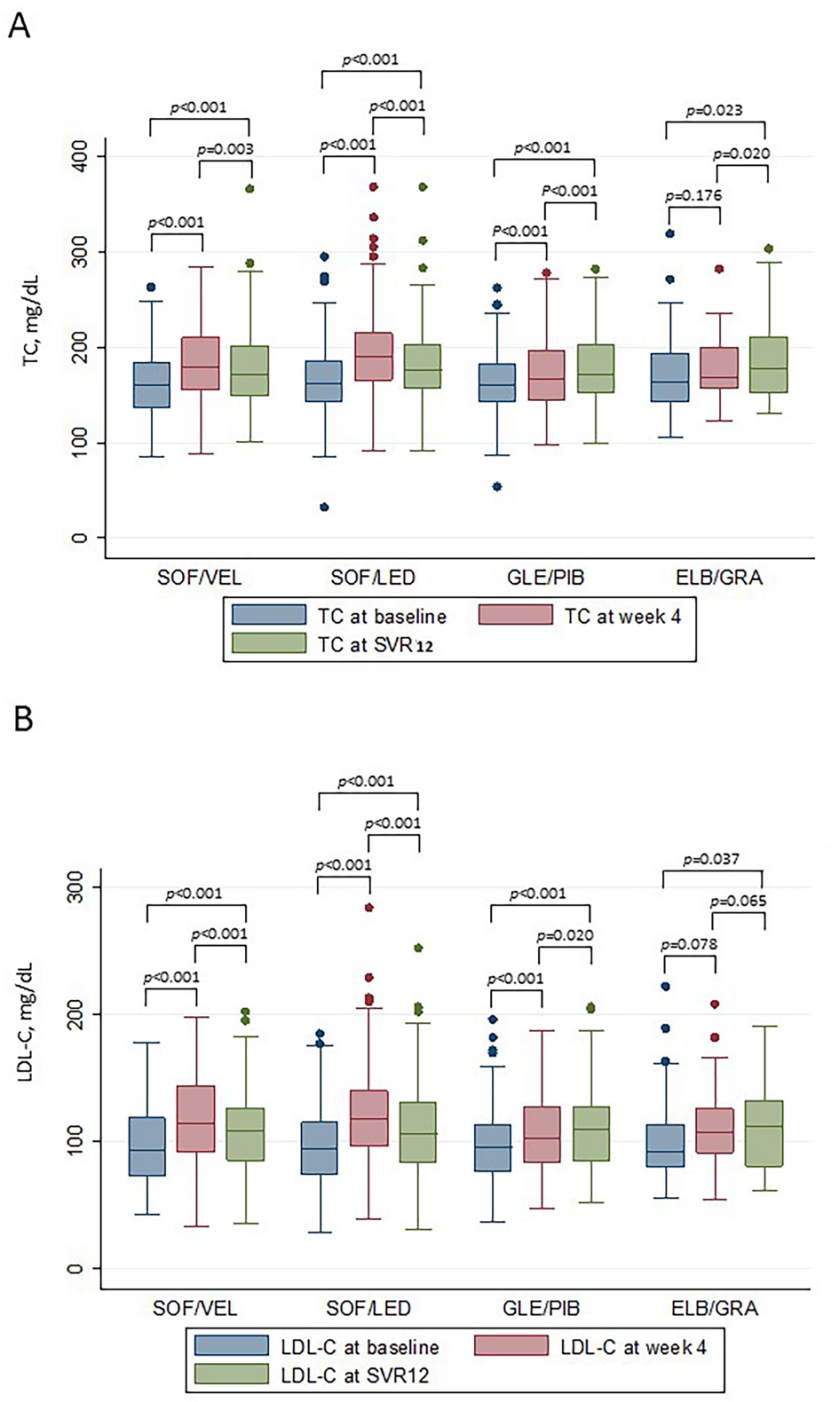
Boxplots showing total cholesterol (TC) (**A**) and LDL-C (**B**) levels at baseline, week-4 and SVR12 during different DAAs treatment. Patient number: SOF/VEL (n = 124), SOF/LED (n = 206), GLE/PIB (n = 122) and ELB/GRA (n = 35). TC and LDL-C levels at baseline, week-4 and SVR12 were compared by Wilcoxon matched-pairs signed rank test.

Three hundred and thirty patients achieving SVR12 were treated with SOF-based regimens (124 SOF/VEL and 206 SOF/LED), and 157 patients were treated with non-SOF-based (122 GLE/PIB and 35 ELB/GRA) with or without ribavirin (8 patients in SOF/VEL and 7 patients in SOF/LED) for 8 or 12 weeks as shown in Table 1 (DAA regimens). Ribavirin groups were treated for 12 weeks. At week 4, elevated TC (Fig. 2A) and LDL-C (Fig. 2B) were noted in patients treated with SOF/VEL (*p* < 0.001), SOF/LED (*p* < 0.001) and GLE/PIB (*p* < 0.001), but not in patients treated with ELB/GRA (TC, *p* = 0.176; LDL-C, *p* = 0.078). The elevations were sustained at SVR12 with SOF/VEL, SOF/LED and GLE/PIB (*p* < 0.001). However, the ELB/GRA group showed higher TC and LDL-C at SVR12 than baseline (TC, *p* = 0.023; LDL-C, *p* = 0.037).

### Amplitudes of changes in TC and LDL-C between SOF- and non-SOF-based regimens

**Figure 3:**
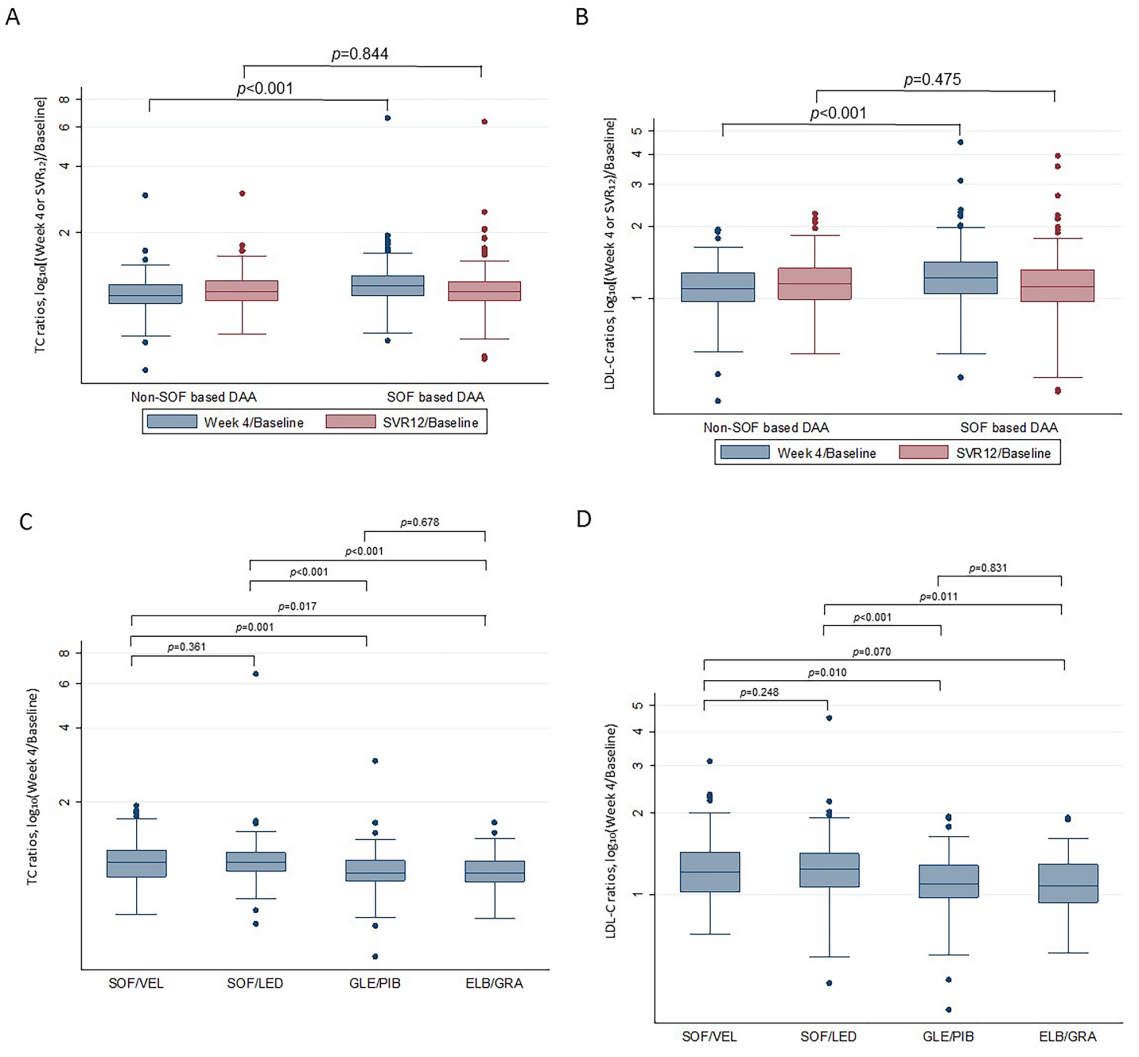
Boxplots showing TC and LDL-C ratios of week 4/baseline and SVR12/baseline in SOF-based and non-SOF-based DAA (TC in **A**; LDL-C in **B**) and in different DAA regimens (TC in **C**; LDL-C in **D**). The Y-axis scale is the value of log_10_[(Week 4 or SVR_12_)/Baseline]. The differences were compared by Mann–Whitney's test.

Amplitudes of changes in serum TC and LDL-C from baseline to week 4 (or SVR12) were expressed as Log_10_ [(Week 4 or SVR12)/Baseline] and shown at Y axis of Fig. 3. SOF-based regimens caused significantly larger amplitude of change in TC (Fig. 3A, p < 0.001) and LDL-C (Fig. 3B, p < 0.001) at week 4 compared to non-SOF-based regimens. These changes were not sustained at SVR12 (TC, *p* = 0.884; LDL-C, *p* = 0.475). The stratified analyses compared different two regimens and showed that there was no significant difference in log_10_ (Week4/Baseline) change of both TC (Fig. 3C, p = 0.361) and LDL-C (Fig. 3D, p = 0.248) between the two SOF-based regimens, SOF/VEL and SOF/LED. The statistically significant changes in TC and LDL-C were noted at these two-regimen comparisons: SOF/VEL vs GLE/PIB (TC, *p* = 0.001; LDL-C, *p* = 0.010), SOF/LED vs GLE/PIB (TC, *p* < 0.001; LDL-C, *p* < 0.001), and SOF/LED vs ELB/GRA (TC, *p* < 0.001; LDL-C, *p* = 0.011). The *p* value of TC at SOF/VEL vs ELB/GRA was 0.017, and that of LDL-C was 0.070. These data suggested that SOF was associated with the major inductor of higher week-4 lipid change.

### Incidence comparisons of increased TC or LDL-C in SOF- and non-SOF-based regimens

To make TC and LDL-C elevations easy to understand, we classified week-4 TC and LDL-C changes to increases > 10% ([Week4/Baseline] > 1.1) and > 25% ([Week4/Baseline] > 1.25) by patient numbers (Table 3). The SOF-based regimens showed higher incidences of increases in TC and LDL-C > 10% (TC, *p* < 0.001; LDL-C, *p* < 0.001) or > 25% (TC, *p* = 0.003; LDL-C, *p* = 0.001) (Table 3). Relative risk (RR) of TC elevation > 10% for SOF-based regimens was 2.72-fold higher than non-SOF-based regimens (95% CI 1.84–4.02, *p* < 0.001), and that of LDL-C was 2.04 (95% CI 1.39–3.01, *p* < 0.001) (Table 4). RR of TC elevation > 25% for SOF-based regimens was 2.11-fold higher than non-SOF-based regimens (95% CI 1.28–3.47, *p* = 0.003), and that of LDL-C was 2.04 (95% CI 1.36–3.06, *p* = 0.001) (Table 4).

**Table 3:** The incidence of 10% or 25% increase in total cholesterol or LDL-C at week 4 in CHC patients treated with SOF- or non-SOF-based regimens (n = 487).

DAAs regimens	Total cholesterol				LDL-C			
	> 10%		> 25%		> 10%		> 25%	
	No (%)	*p*	No (%)	*p*	No (%)	*p*	No (%)	*p*
Sofosbuvir-based regimen (n = 330)	211 (63.9)	< 0.001	91 (27.6)	0.003	215 (65.2)	< 0.001	151 (45.8)	0.001
Non-sofosbuvir-based regimen (n = 157)	62 (39.5)		24 (15.3)		75 (47.8)		46 (23.9)	

DAAs: direct-acting antivirals.

**Table 4:** The relative risk of total cholesterol and LDL-C ratio > 1.10 or 1.25 in CHC patients treated with DAA regimens at week 4 (n = 487).

DAA regimens	Total cholesterol ratio				LDL-C ratio			
	Week 4/baseline > 1.10		Week 4/baseline > 1.25		Week 4/baseline > 1.10		Week 4/baseline > 1.25	
	RR (95%CI)	*p*	RR (95%CI)	*p*	RR (95%CI)	*p*	RR (95%CI)	*p*
SOF-based regimen (n = 330) vs non-SOF-based regimen (n = 157)	2.72 (1.84–4.02)	< 0.001	2.11 (1.28–3.47)	0.003	2.04 (1.39–3.01)	< 0.001	2.04 (1.36–3.06)	0.001
SOF/VEL (n = 124) vs SOF/LED (n = 206)	0.75 (0.47–1.18)	0.212	1.28 (0.78–2.09)	0.334	0.68 (0.43–1.08)	0.106	0.74 (0.47–1.16)	0.191
SOF/VEL (n = 124) vs GLE/PIB (n = 122)	2.13 (1.28–3.54)	0.004	2.40 (1.29–4.46)	0.006	1.63 (0.99–2.71)	0.057	1.74 (1.02–2.95)	0.042
SOF/LED (n = 206) vs GLE/PIB (n = 122)	2.86 (1.80–4.54)	< 0.001	1.88 (1.05–3.36)	0.033	2.39 (1.51–3.80)	< 0.001	2.35 (1.45–3.78)	< 0.001
SOF/VEL (n = 124) vs ELB/GRA (n = 35)	2.84 (1.29–6.22)	0.009	2.65 (0.96–7.36)	0.061	1.57 (0.74–3.33)	0.243	1.52 (0.69–3.39)	0.301
SOF/LED (n = 206) vs ELB/GRA (n = 35)	3.81 (1.79–8.10)	0.001	2.08 (0.77–5.63)	0.150	2.30 (1.11–4.74)	0.025	2.06 (0.96–4.42)	0.064
GLE/PIB (n = 122) vs ELB/GRA (n = 35)	1.33 (0.61–2.92)	0.476	1.11 (0.38–3.21)	0.852	0.96 (0.45–2.04)	0.914	0.88 (0.39–1.98)	0.754

DAAs: direct-acting antivirals, ELB/GRA: elbasvir/grazoprevir, GLE/PIB: glecaprevir/pibrentasvir, SOF/LED: sofosobuvir/ledipasvir, SOF/VEL: sofosbuvir/velpatasvir, RR: relative risk.

There were no significant differences in week-4 TC and LDL-C elevations between two SOF regimens (SOF/VEL and SOF/LED) and between two non-SOF regimens (GLE/PIB and ELB/GRA) in either increase > 10% or > 25%. However, in TC > 10%, all SOF vs non-SOF (SOF/VEL vs GLE/PIB, SOF/VEL vs ELB/GRA, SOF/LED vs GLE/PIB, and SOF/LED vs ELB/GRA) showed significant differences in TC elevation. In LDL-C > 10%, SOF/LED caused significant higher risk in LDL-C elevation than GLE/PIB and ELB/GRA. In TC or LDL-C > 25%, SOF-based regimen still showed significant higher risk in some SOF vs non-SOF paired comparisons (SOF/VEL vs GLE/PIB and SOF/LED vs GLE/PIB). In TC or LDL-C > 25%, there are no difference in comparisons SOF/VEL vs ELB/GRA, and SOF/LED vs ELB/GRA. These statistics gave further evidence for SOF in worsened week-4 lipid profiles. The SOF-based lipid-elevation effects were not seen at SVR12 (Supplementary Table 1).

### Multivariable logistic regression for possible confounders

The variables in Table 1, which affect SOF- and non-SOF-based statistics (p < 0.05) were subjected to multivariable logistic regression which evaluates confounders of HIV infection, WBC, platelet, DB, AST, creatinine, eGFR, hemodialysis and F4 status, and also classified into week 4/baseline > 1.10 and > 1.25 for TC and LDL-C as shown in supplementary Table 2 (sTable 2). SOF vs non-SOF still gave significant differences in TC and LDL-C at week 4/baseline either > 1.10 or > 1.25. Baseline WBC was negatively associated with TC elevation > 10% at week 4 (adjusted RR = 0.99, *p* = 0.033). Baseline AST was positively associated with LDL-C elevation > 25% at week 4 (adjusted RR = 1.01, *p* = 0.021). Hemodialysis was negatively associated with TC and LDL-C elevations either > 10% or > 25% (all with adjusted RR and *p* < 0.05). The influence of WBC, AST, and hemodialysis on TC and LDL-C during DAAs treatment is uncertain and requires further study to clarify relationship between them.

### Chronic diseases-stratified analysis in SOF- and non-SOF groups

To determine whether chronic diseases affected TC and LDL-C during DAA treatment and at SVR12, we stratified SOF and non-SOF groups into disease subgroups including cardiovascular disease, hypercholesterolemia, diabetes mellitus, and chronic kidney disease (sTable 3). Patients with cardiovascular disease and chronic renal disease had higher percentage of patients taking non-SOF-based regimens (sTable 3, *p* values). The disease-stratified analysis showed that patients with hypercholesterolemia and diabetes mellitus did not have significant elevation of TC and LDL-C (> 10%) at week 4 (sTable 4) and SVR12 (sTable 5). Patients with cardiovascular disease showed elevated TC (*p* = 0.004) and LDL-C (*p* = 0.035) at week 4 (sTable 4) and elevated LDL-C (*p* = 0.027) at SVR12 (sTable 5). Patients with chronic kidney disease showed elevated LDL-C at week 4 (*p* = 0.040, sTable 4). These data suggest that patients with normal baseline TC and LDL-C contributed more to elevations of TC and LDL-C at week 4 than those with baseline hypercholesterolemia. It is known that diabetes mellitus patients are prone to have elevated blood cholesterol. Then we divided both SOF- and non-SOF groups into normal baseline cholesterol and baseline hypercholesterolemia subgroups. As shown in sTable 6, patients with normal baseline TC and LDL-C showed significant increases in TC (*p* < 0.001) and LDL-C (*p* = 0.001) at week 4, but the elevations were not sustained to SVR12 (sTable 7), consistent with data shown in Fig. 3A,B. SOF did not cause significant change in TC and LDL-C at week 4 (sTable 6) and SVR12 (sTable 7) in hypercholesterolemia subgroups.

## Discussion

DAAs have been shown to worsen lipid profiles during CHC treatment. To the best of our knowledge, this study is the first retrospective analysis to evaluate the effect of SOF-based DAAs on changes in lipid profiles. Our results showed that compared with non-SOF DAAs, SOF-based DAAs were associated with significant increases in TC and LDL-C during the initial 4 weeks of treatment. However, the SOF-potentiated effects were not sustained at the end of treatment. A further comparison of SOF/VEL or SOF/LED with GLE/PIB and ELB/GRA revealed a similar trend. It is interesting to note that ELB/GRA did not cause TC and LDL-C elevation at week 4. Very limited literature documented association of ELB/GRA with lipid worsening during CHC treatment. Sun et al*.* reported 24 cases, 13 treated with ELB/GRA and 11 with SOF/LED, showing significant elevation of cholesterol at week 4^25^, which might be the effect of SOF/LED rather than ELB/GRA because our data showed that ELB/GRA did not cause significant increase in total cholesterol at week 4. However, at SVR12, TC and LDL-C increased in ELB/GRA group compared with its baseline (TC, *p* = 0.023; LDL-C, *p* = 0.037, Fig. 2).

Combination DAAs have been the treatment standard for CHC. Our data provide strong evidence that SOF-based DAAs resulted in higher elevation of TC and LDL-C than non-SOF-based regimens at week 4 after drug administration (Table 3, Figs. 2 and 3). Since the two kinds of regimens achieved similar SVR12, viral clearance appeared not to be the sole mechanism to account for different TC and LDL-C changes between the two. We compared three compounds, sofosbuvir, tenofovir alafenamide (TAF), and tenofovir disoproxil fumarate (TDF) (Fig. 1) and found that SOF and TAF have similar phosphoramidate side chains (Fig. 1A, colored structures in SOF and TAF) and both cause elevated blood cholesterol in the current (SOF) and previous studies (TAF)^19–21^. However, TDF without the side chains did not affect cholesterol level. Milinkovic et al. reported changes in lipid profile in HIV patients treated with TDF or TAF. After switching from TDF to TAF, mean total cholesterol increased from 186 ± 37 mg/dL at baseline to 206 ± 43 mg/dL and 204 ± 43 mg/dL at weeks 12 and 24 (*p* < 0.001), and the increase in total cholesterol was mainly due to an increase in LDL-C^20^. Similar lipid changes were reported by other HIV investigators^21,26^.

For CHC patients, SOF-based DAAs significantly elevated TC and LDL-C levels during the initial 4 weeks of treatment, but the higher amplitudes of changes in TC and LDL-C tended to disappear at the end of treatment. It could be deduced from the chemical structures of SOF and TAF that the cleaved products phenolate ion and propan-2-yl 2-aminopropanoate from the phosphoramidate side chain may promote β-lipoprotein synthesis and secretion initially and then induce more enzymes to destroy them with time, so TC and LDL-C levels jump transiently and then decline. SOF-based DAA regimens have been shown to have cardiotoxicity^27,28^, so careful monitoring of initial four weeks of lipid profiles is important, especially for patients with ischemic heart disease or diabetes mellitus.

Our results showed trends of increases in TC and LDL-C at week 4 and SVR12. Some reports showed the increases in TC or LDL-C disappeared after treatment^29,30^. Other studies suggested the elevated LDL-C continued to post-treatment 1 year^31–34^. The main reason for the inconsistency was that most of these studies were single-arm studies with different drugs and doses and without placebo controls. Additionally, both Younossi et al. and Pedersen et al. observed that genotype 3 patients had significantly increased LDL-C during DAAs treatment, but genotype 1 or genotype 2 patients did not^35,36^. As the majority of the included patients in the present study were genotype 1 (59.6%) and genotype 2 (32.9%) patients (Table 1), changes in LDL-C would be less, consequently reducing the difference between treatment groups. Furthermore, genetic factors have been reported in association with changes in LDL-C. In the study by Emmanuel et al., the *IFNL4-ΔG* carriers had significant increases in LDL-C during DAAs treatment and at post-treatment 1 year, but the patients with *IFNL4-TT/TT* did not^37^. In the study by Morihana et al., the difference in LDL-C between sofobusvir/ledipasvir and daclatasvir/asunaprevir disappeared after the end of treatment. However, the *IL28B TG/GG* patients continued to have increased LDL-C from the end of treatment to post-treatment 2 years, whereas the *IL28B TT* patients did not^29^. Because the current analysis did not consider these genetic factors, our results might be potentially confounded by these predictors.

Although it was common to observe increases in TC and LDL-C during DAAs treatment, a few studies analyzing risk factors for increased LDL-C during DAAs treatment revealed discordant findings. In the study by Morihana et al., multivariate regression analysis showed that changes in LDL-C were negatively correlated with baseline LDL-C and HDL^29^. Hashimoto et al. reported that the decline of HCV core antigen from day 0 to day 1 was independently associated with amplitudes of changes in LDL-C but not with baseline LDL-C during DAA treatment^38^. The discordance highlighted the need of prospective large-scale long-term studies to elucidate the effect of DAAs on lipid profiles.

There were several limitations in this study. Firstly, lipid profiles are incomplete without HDL and triglyceride. Secondly, case numbers are uneven among DAA regimens. The third is that the included patients in this study were mainly those who achieved SVR12. In addition, we did not have lipid data of post-treatment long-term follow-up. Therefore, it deserves more comparative studies, which evaluate complete lipid profiles at different time points among DAAs regimens. In conclusion, chemical structure of DAAs may be one of the mechanisms causing hyperlipidemia during anti-HCV treatment in addition to abrupt lipoprotein release from hepatocyte to the blood during HCV clearance. SOF contributes more to dyslipidemia than other DAAs probably on account of its phosphoramidate side chain, which deserves attention in patients taking drugs containing the side chain long term such as TAF to treat HIV and HBV. Dyslipidemia may increase cardio- and cerebro-vascular events in these patients.

## Supplementary Information

Supplementary Tables.

## Data Availability

The datasets generated during and analyzed during the current study are not publicly available but are available from the corresponding author on reasonable request.
