# Supplementary material for: Sofosbuvir-based direct-acting antivirals and changes in cholesterol and low density lipoprotein-cholesterol
Source: Sci Rep. 2022 Jun 15;12:9942. doi: 10.1038/s41598-022-13657-5 (PMC9200852; doi:10.1038/s41598-022-13657-5)
Supplement: Supplementary file 1 — Supplementary Tables. [file 41598_2022_13657_MOESM1_ESM.pdf]

# Supplementary data

**Table 1. The relative risk of cholesterol and LDL ratio >1.10 in CHC patients treated with DAA regimens at $SVR_{12}$ (n=487)**

| | Cholesterol ratio | | LDL ratio | |
|---|---|---|---|---|
| | SVR12/baseline >1.10 | | SVR12/baseline >1.10 | |
| DAA regimen | RR (95%CI) | *p* | RR (95%CI) | *p* |
| Sofosbuvir-based regimen (n=330) vs Non-Sofosbuvir-based regimen (n=157) | 0.90 (0.61–1.32) | 0.584 | 0.93 (0.64–1.36) | 0.713 |
| SOF/VEL (n=124) vs SOF/LED (n=206) | 1.07 (0.68–1.67) | 0.774 | 0.97 (0.62–1.52) | 0.908 |
| SOF/VEL (n=124) vs GLE/PIB (n=122) | 0.97 (0.59–1.60) | 0.904 | 1.30 (0.63–1.70) | 0.904 |
| SOF/LED (n=206) vs GLE/PIB (n=122) | 0.91 (0.58–1.42) | 0.674 | 1.06 (0.68–1.66) | 0.803 |
| SOF/VEL (n=124) vs ELB/GRA (n=35) | 0.83 (0.39–1.76) | 0.627 | 0.59 (0.27–1.30) | 0.191 |
| SOF/LED (n=206) vs ELB/GRA (n=35) | 0.78 (0.38–1.59) | 0.491 | 0.61 (0.29–1.29) | 0.196 |
| GLE/PIB (n=122) vs ELB/GRA (n=35) | 0.86 (0.40–1.82) | 0.685 | 0.58 (0.26–1.26) | 0.167 |

**Table 2. The multivariable logistic regression of total cholesterol and LDL-C ratio >1.10 and 1.25 in CHC patients at week 4**

| | Total cholesterol ratio | | | | LDL-C ratio | | | |
|---|---|---|---|---|---|---|---|---|
| | Week 4/baseline >1.10 | | Week 4/baseline >1.25 | | Week 4/baseline >1.10 | | Week 4/baseline >1.25 | |
| Variables | Adj RR (95%CI) | *p* | Adj RR (95%CI) | *p* | Adj RR (95%CI) | *p* | Adj RR (95%CI) | *p* |
| Sofosbuvir-based DAAs (yes vs no) | 2.83 (1.85–4.34) | <0.001 | 2.43 (1.41–4.21) | 0.001 | 1.91 (1.25–2.91) | 0.003 | 2.00 (1.29–3.11) | 0.002 |
| HIV (positive vs negative) | 1.09 (0.52–2.25) | 0.822 | 0.53 (0.21–1.37) | 0.195 | 1.24 (0.60–2.57) | 0.564 | 1.04 (0.51–2.15) | 0.905 |
| WBC (baseline) | 0.99 (0.99–0.99) | 0.033 | 1.00 (0.99–1.00) | 0.593 | 0.99 (0.99–1.00) | 0.304 | 1.00 (0.99–1.00) | 0.942 |
| Platelet (baseline) | 1.00 (0.99–1.00) | 0.349 | 1.00 (0.99–1.00) | 0.723 | 1.00 (0.99–1.00) | 0.974 | 1.00 (0.99–1.00) | 0.858 |
| Direct bilirubin (baseline) | 1.10 (0.83–1.45) | 0.505 | 0.95 (0.63–1.44) | 0.825 | 1.05 (0.80–1.38) | 0.701 | 0.75 (0.39–1.45) | 0.393 |
| AST (baseline) | 1.00 (0.99–1.01) | 0.105 | 1.00 (0.99–1.01) | 0.279 | 1.00 (0.99–1.01) | 0.066 | 1.01 (1.01–1.01) | 0.021 |
| Creatinine (baseline) | 1.28 (0.96–1.70) | 0.093 | 1.37 (0.97–1.94) | 0.075 | 1.38 (0.98–1.93) | 0.064 | 1.31 (0.96–1.79) | 0.089 |
| eGFR (baseline) | 1.00 (0.99–1.01) | 0.679 | 1.00 (0.99–1.01) | 0.995 | 0.99 (0.99–1.01) | 0.931 | 1.00 (0.99–1.01) | 0.673 |
| eGFR ≥60 mL/min/1.73m$^2$ (yes vs no) | 0.89 (0.41–1.93) | 0.766 | 0.68 (0.29–1.58) | 0.370 | 0.90 (0.42–1.97) | 0.801 | 0.81 (0.38–1.74) | 0.590 |
| Hemodialysis (yes vs no) | 0.10 (0.01–0.85) | 0.034 | 0.04 (0.01–0.74) | 0.031 | 0.02 (0.01–0.36) | 0.007 | 0.07 (0.01–0.85) | 0.037 |
| F4 fibrosis (yes vs no) | 0.73 (0.35–1.51) | 0.394 | 0.66 (0.28–1.56) | 0.345 | 0.78 (0.38–1.64) | 0.517 | 0.94 (0.45–1.94) | 0.857 |

**Table 3. Chronic disease stratification of SOF- and non-SOF groups**

| | All patients (n=487) | Patients with SOF-based regimen (n=330) | Patients with Non-SOF-based regimen (n=157) | *p* # |
|---|---|---|---|---|
| Cardiovascular diseases, (%) | 99 (20.3) | 57 (17.3) | 42 (26.8) | 0.015 |
| Hypercholesterolemia, (%) | 85 (17.5) | 56 (17) | 29 18.5) | 0.703 |
| (TC ≥200 mg/dL and/or LDL ≥130 mg/dL) | | | | |
| Diabetes mellitus, (%) | 65 (13.4) | 40 (12.1) | 25 (15.9) | 0.257 |
| Chronic kidney disease , (%) | 48 (9.9) | 21 (6.4) | 27 (17.2) | <0.001 |
| (eGFR ≤45mL/min/1.73m$^2$) | | | | |

#SOF-based regimen versus Non-SOF-based regimen.
Categorical variables are expressed as number of patients (n) with frequencies (%) analyzed by Chi-squared test and Fisher's exact test.

**Table 4. The incidence of 10% increase in TC or LDL-C at week 4 in CHC patients treated with SOF- or non-SOF-based regimens by disease subgroups**

| | Total cholesterol >10% at week 4 | | | LDL-C >10% at week 4 | | |
|---|---|---|---|---|---|---|
| | SOF-based regimens | non-SOF-based regimens | | SOF-based regimens | non-SOF-based regimens | |
| Disease subgroups | n/N (%) | n/N (%) | *p* | n/N (%) | n/N (%) | *p* |
| Cardiovascular diseases | 39/57 (68.4) | 16/42 (38.1) | 0.004 | 42/57 (73.7) | 22/42 (52.4) | 0.035 |
| Hypercholesterolemia (TC≥200 mg/dL and/or LDL≥130 mg/dL) | 23/56 (41.2) | 8/29 (27.6) | 0.245 | 21/56 (37.5) | 6/29 (20.7) | 0.144 |
| Diabetes mellitus | 25/40 (62.5) | 12/25 (48) | 0.307 | 30/40 (75) | 14/25 (56) | 0.172 |
| Chronic kidney disease (eGFR≤45mL/min/1.73m$^2$) | 14/21 (66.7) | 11/27 (40.7) | 0.089 | 14/21 (66.7) | 9/27 (33.3) | 0.040 |

N: total number of the subgroup; n: number of TC or LDL-C increase larger than 10%.

**Table 5. The incidence of 10% increase in TC or LDL-C at SVR12 in CHC patients treated with SOF- or non-SOF-based regimens by disease subgroups**

| | Total cholesterol >10% at SVR12 | | | LDL-C >10% at SVR12 | | |
|---|---|---|---|---|---|---|
| | SOF-based regimens | non-SOF-based regimens | | SOF-based regimens | non-SOF-based regimens | |
| Disease subgroups | n/N (%) | n/N (%) | *p* | n/N (%) | n/N (%) | *p* |
| Cardiovascular diseases | 21/57 (36.8) | 21/42 (50) | 0.221 | 25/57 (43.9) | 28/42 (66.7) | 0.027 |
| Hypercholesterolemia (TC≥200 mg/dL and/or LDL≥130 mg/dL) | 8/56 (14.3) | 8/29 (27.6) | 0.154 | 11/56 (19.6) | 9/29 (31) | 0.285 |
| Diabetes mellitus | 18/40 (45) | 14/25 (56) | 0.450 | 20/40 (50) | 14/25 (56) | 0.799 |
| Chronic kidney disease (eGFR≤45mL/min/1.73m$^2$) | 11/21 (52.4) | 14/27 (51.9) | 1.000 | 11/21 (52.4) | 16/27 (59.3) | 0.771 |

N: total number of the subgroup; n: number of TC or LDL-C increase larger than 10%.

**Table 6. The effect of cholesterol level on the incidence of 10% increase in TC or LDL-C at week 4 in CHC patients treated with SOF- or non-SOF-based regimens**

| | TC >10% at week 4 | | | LDL-C >10% at week 4 | | |
|---|---|---|---|---|---|---|
| | SOF-based regimens | non-SOF-based regimens | | SOF-based regimens | non-SOF-based regimens | |
| **Disease subgroups** | **n/N (%)** | **n/N (%)** | ***p*** | **n/N (%)** | **n/N (%)** | ***p*** |
| **Normal cholesterol level (TC <200 mg/dL and LDL <130 mg/dL)** | **188/274 (68.6)** | **54/128 (42.2)** | **<0.001** | **194/274 (70.8)** | **69/128 (53.9)** | **0.001** |
| **Hypercholesterolemia (TC ≥200 mg/dL and/or LDL ≥130 mg/dL)** | **23/56 (41.2)** | **8/29 (27.6)** | **0.245** | **21/56 (37.5)** | **6/29 (20.7)** | **0.144** |

**N: total number of the subgroup; n: number of TC or LDL-C increase larger than 10%.**

**Table 7. The effect of cholesterol level on the incidence of 10% increase in TC or LDL-C at SVR12 in CHC patients treated with SOF- or non-SOF-based regimens**

| | TC >10% at SVR12 | | | LDL-C >10% at SVR12 | | |
|---|---|---|---|---|---|---|
| | SOF-based regimens | non-SOF-based regimens | | SOF-based regimens | non-SOF-based regimens | |
| **Disease subgroups** | **n/N (%)** | **n/N (%)** | ***p*** | **n/N (%)** | **n/N (%)** | ***p*** |
| **Normal cholesterol level (TC <200 mg/dL and LDL <130 mg/dL)** | **143/274 (52.2)** | **68/128 (53.1)** | **0.861** | **166/274 (60.6)** | **78/128 (60.9)** | **0.946** |
| **Hypercholesterolemia (TC ≥200 mg/dL and/or LDL ≥130 mg/dL)** | **8/56 (14.3)** | **8/29 (27.6)** | **0.154** | **11/56 (19.6)** | **9/29 (31)** | **0.285** |

**N: total number of the subgroup; n: number of TC or LDL-C increase larger than 10%.**
